# Successful Ocular Structural Restoration of Aggressive Necrotizing Sclerouveitis Following Pterygium Excision: Infectious, Autoimmune, or Both?

**DOI:** 10.21203/rs.3.rs-9954548/v1

**Published:** 2026-06-22

**Authors:** Cristian de los Santos, David Chanthan, Dalia El-Feky, Gunjan Awatramani, Amir Akhavanrezayat, Alan Sherif, Negin Yavari, David Ni Xu, Aubrey Nguyen, Isaac Sanchez, Osama Elaraby, Jingli Guo, Bin Mo, Ishaan Iyer, Mohammad Rajabi, Azadeh Mobasserian, Ahn Ngoc Tram Tran, Yu-Kuei Lee, Gunay Uludag Kirimli, Ngoc Trong Tuong Than, Jia-Horung Hung, Quan Dong Nguyen

**Affiliations:** Byers Eye Institute; Khmer-Soviet Friendship Hospital; Byers Eye Institute; Byers Eye Institute; Byers Eye Institute; Byers Eye Institute; Byers Eye Institute; Byers Eye Institute; Byers Eye Institute; Byers Eye Institute; Byers Eye Institute; Byers Eye Institute; Byers Eye Institute; Byers Eye Institute; Byers Eye Institute; Byers Eye Institute; Byers Eye Institute; Byers Eye Institute; Byers Eye Institute; Byers Eye Institute; Byers Eye Institute; Byers Eye Institute

**Keywords:** Necrotizing scleritis, surgically induced scleral necrosis, pterygium excision, sclero-uveitis, infection, autoimmune, rituximab

## Abstract

**Purpose:**

To report a case of severe refractory surgically induced scleral necrosis (SISN) with pan-uveitis and retinal detachment following pterygium excision, highlighting the challenges in differentiating infectious versus autoimmune mechanisms and demonstrating successful restoration of ocular structure with combined surgical and immunosuppressive therapy in a patient with poor initial prognosis.

**Observation:**

An 80-year-old man presented one year after pterygium surgery with severe pain, redness, and vision loss in the right eye. Examination revealed nasal necrotizing scleritis with purulent discharge, extensive scleral thinning and uveal exposure, pan-uveitis, serous retinal detachment, and choroidal folds. Co-infection was suspected, and systemic autoimmune predisposition was suggested by p-ANCA positivity and history of cutaneous lupus. The patient underwent urgent scleral debridement and biopsy, along with topical and systemic antibiotics, followed by weekly rituximab infusions and mycophenolate mofetil under steroid taper. After eight cycles of active treatment, scleral inflammation improved significantly, scleral thickness restored, and retinal detachment and subretinal fluid completely resolved. At last follow-up, visual acuity improved from counting fingers to 20/100 with restoration of ocular structural integrity.

**Conclusion:**

The index case highlights the challenge of distinguishing infection from autoimmunity in SISN. Early debridement, prompt antimicrobial treatment, and immunosuppressive therapy are crucial in the management of SISN. Rituximab infusion is safe and effective for rapidly controlling severe scleral inflammation once infection is ruled out or is under control.

## 1. Introduction

Scleritis is an uncommon and potentially sight-threating inflammation of sclera that can extend to adjacent ocular tissues.^1^ Watson and Hayreh anatomically classified scleritis into anterior and posterior types with anterior scleritis further subdivided clinically into diffuse, nodular and necrotizing forms, the latter occurring with or without inflammation (scleromalacia perforans).^2^ The severity grading system was established by Sen et. al using standard clinical images after 10% phenylephrine eyedrops and ranges from 0 to 4 + grade.^3^

Among all subtypes, necrotizing scleritis is considered the most severe, given progressive scleral destruction and high risk of vision loss. Scleral necrosis can occur following surgical trauma, termed surgically induced scleral necrosis (SISN), either in isolation or in association with infection or systemic autoimmune disease.^4,5^ The onset of SISN is highly variable, ranging from the first postoperative day to more than 50 years after an ocular procedure. It has been reported following a wide range of interventions, including pterygium excision (with or without mitomycin C or beta radiation), conjunctival excision, cataract extraction, strabismus surgery, glaucoma surgery, and vitreoretinal surgery. SISN has also been reported after transscleral cyclodiode or cryotherapy procedures, and intravitreal injection.^4^

Infectious SISN is more frequently reported after pterygium surgery.^6^ However, an underlying systemic autoimmune disease should be considered even when an infectious etiology is established, as it is documented in 32–57% of scleritis patients.^4^ Therefore, comprehensive infectious and immunological work-up must be performed.^4–10^

Excluding an infectious etiology can be challenging, resulting in delayed treatment and poor outcomes. Moreover, the prognosis may deteriorate when corticosteroids are inadvertently administered without antimicrobial coverage under the assumption of immune-mediated scleritis.^7,8^ The management of SISN involves antimicrobial therapy, nonsteroidal anti-inflammatory drugs, systemic steroids, aggressive immunosuppressive therapy with surgical intervention for severe scleral thinning or perforation.^6,7,11^

Herein, we report a case of an 80-year-old man who developed SISN associated with posterior scleritis and uveitis one year following a pterygium surgery in the right eye (OD), presenting with severe pain, redness, and blurred vision, requiring surgical debridement and pus drainage, aggressive immunosuppressive therapy with a combination of rituximab infusions, mycophenolate mofetil under the umbrella of fortified antibiotic therapy for suspect bacterial coinfection.

## 2. Case Report

An 80-year-old Caucasian male presented with redness and pain in OD since early June 2025 and was initially diagnosed with anterior uveitis, for which he received topical steroids and cycloplegics. Despite treatment, his ocular pain worsened, accompanied by photophobia and blurred vision. Diagnosis of sclerouveitis in OD was made, and he was referred to our tertiary eye center in July 2025. Ocular history was notable for nasal pterygium excision in November 2024, although the surgical technique was not specified. Medical history was significant for type 2 diabetes mellitus, cutaneous lupus erythematous, coronary artery disease, and atrial fibrillation.

**Figure 1:**
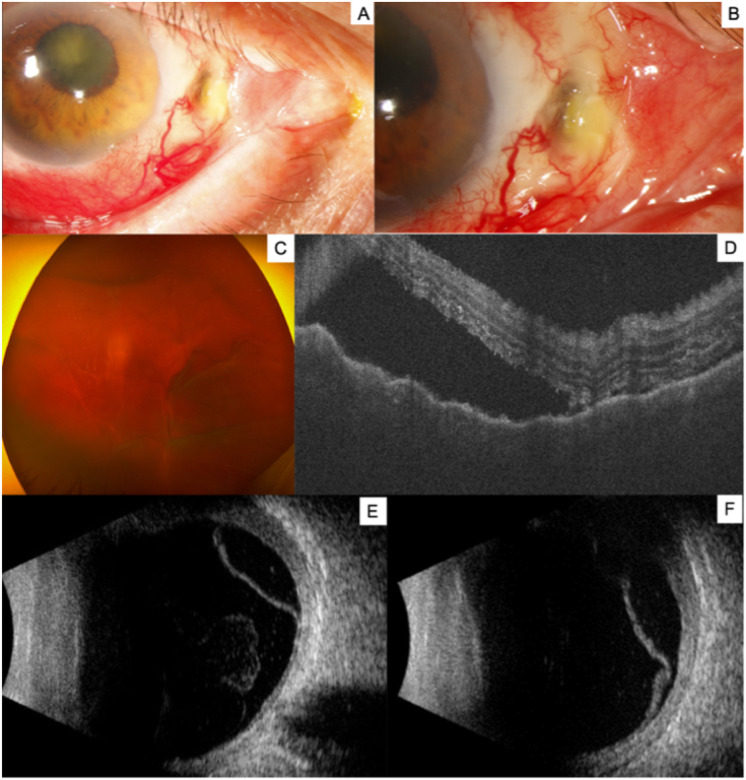
Initial presentation. A. Slit-lamp photos of the right eye (OD) demonstrate nasal 3 clock-hour ischemic scleral thinning and excavation with uveal show, purulent appearance, and diffuse redness of the remaining sclera. Posterior synechiae and cataractous lens were also noted. C. Fundus photo OD reveals a hazy view and serous retinal detachment. D. SD-OCT OD illustrates sub-retinal fluid involving the fovea with choroidal folds, subretinal precipitates. E-F. B-scan OD with dense, heterogeneous vitreous opacification consistent with vitritis, and underlying choroidal thickening.

On examination of OD, the best-corrected visual acuity (BCVA) was counting fingers with an intraocular pressure (IOP) of 7 mmHg. External examination revealed a 3-clock-hour nasal necrotizing scleritis with a localized 60% scleral thinning with uveal show and overlying purulence, negative Seidel test, and diffuse redness corresponding to + 4 scleral inflammation in the remaining sclera. The slit-lamp examination showed inferior medium-sized keratic precipitates (KPs), 1 + anterior chamber (AC) cells, 360-degree posterior synechiae, and nuclear and posterior subcapsular cataract. (Fig. 1A, B). Fundus examination demonstrated a hazy view, and extensive serous retinal detachment (SRD) involving the macula (Fig. 1C). Spectral-domain optical coherence tomography (SD-OCT) showed choroidal folds, subretinal fluid (SRF) involving the fovea (Fig. 1D). Ultrasound B-scan illustrated heterogeneous vitreous opacity with highly reflective convex retinal detachment and underlying choroidal thickening (Fig. 1E). Left eye (OS) assessment was unremarkable. Hence, the diagnosis of SISN with pan-uveitis was made with suspected *Pseudomonas* co-infection due to the characteristic clinical findings. Therefore, fortified topical vancomycin and tobramycin were prescribed hourly in OD along with oral prednisone (60 mg daily).

A comprehensive systemic work-up revealed neutrophilia and positive perinuclear anti-neutrophil cytoplasmic antibody (p-ANCA). Infection panel results were negative, and chest X-ray was unremarkable. The conjunctival swab culture grew a small number of *Dolosigranulum pigrum*, while viral PCR was negative, and acid-fast bacilli as well as fungal cultures showed no growth.

**Figure 2:**
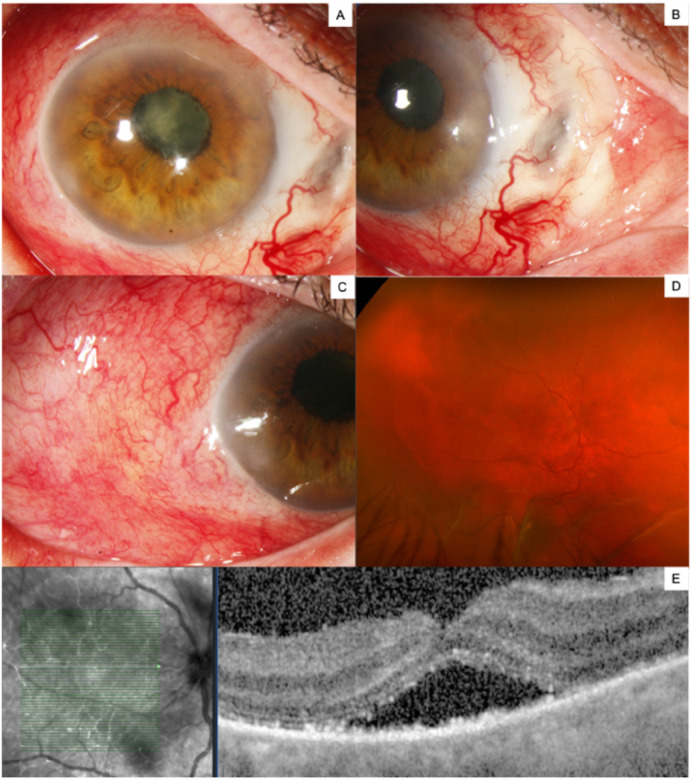
Presentation at subsequent visit. A-C. Slit-lamp photos of the right eye (OD) show nasal scleral thinning with bluish hue and diffuse injection. D. Ultra-wide field fundus photo OD reveals a less hazy view and decreased serous retinal detachment. E. Spectral domain optical coherence tomography OD illustrates resolved choroidal folds, residual minimal sub-foveal fluid, shaggy photoreceptors, and subretinal precipitates, markedly improved compared to the initial presentation.

Two weeks later, at a clinic visit in the Uveitis Service, the patient reported improvement in ocular pain, with stable BCVA and IOP of 5 mmHg. Scleral inflammation improved to + 2 scleral injection, without progression of scleral thinning or purulence, and a yellowish material was observed underneath the temporal conjunctiva, consistent with subconjunctival abscess (Fig. 2A–C). There was no evidence of AC cells or KPs. The fundus examination revealed partial improvement of the SRD (Fig. 2D). SD-OCT showed resolved choroidal folds, minimal residual sub-foveal fluid with disrupted shaggy photoreceptors and subretinal precipitates (Fig. 2E), while OS remained unremarkable.

Given the aggressive presentation with suspected co-infection, urgent conjunctival biopsy with scleral debridement of the purulent tissue was performed. Conjunctival excision revealed a significant amount of purulent material. Sub-tenon and intravenous vancomycin were administered intraoperatively with no additional corticosteroids. Postoperatively, the patient received doxycycline 100 mg twice daily for 7 days and ciprofloxacin 500 mg twice daily for a month. On histopathology, bacterial and fungal cultures, as well as viral PCR, were negative. The patient showed significant improvement; however, due to incomplete resolution of aggressive inflammation, weekly rituximab infusions (375 mg/m^2^ of body surface area) and mycophenolate mofetil (1000 mg twice daily) were initiated under coverage with topical and systemic antibiotics, while oral prednisone was gradually tapered.

**Figure 3:**
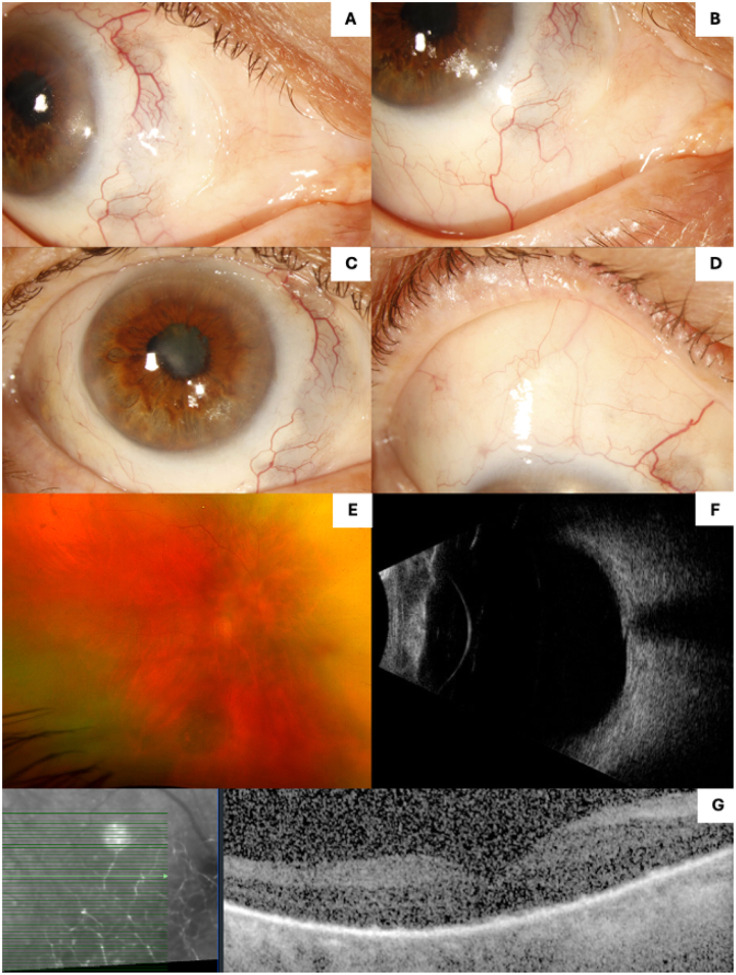
Follow-Up after 8 cycles of rituximab infusions. A-D. Slit-lamp photos of the right eye (OD) demonstrate resolution of scleral injection with healing of SISN with almost no signs of prior excavation and thinning. E. Ultra-wide field fundus photo OD shows no detachment. F. Ultrasound B-scan demonstrates normal choroidal thickening and attached retina. G. Spectral domain optical coherence tomography OD illustrates complete resolution of the subretinal fluid.

At the last follow-up in January 2026, after completing eight weekly rituximab infusions, the patient returned with improved vision (BCVA 20/100), an IOP of 10 mmHg, no scleral injection, improvement in scleral thickness with almost no signs of prior excavation and thinning, and more fine superficial vascularization over the previously avascular sclera in the OD (Fig. 3A–D). On fundus examination, there was no SRD (Fig. 3E), B-scan showed normal choroidal thickening and an attached retina (Fig. 3F), and the SD-OCT confirmed complete resolution of the SRF in the OD (Fig. 3G). The patient continued monthly rituximab infusions and mycophenolate mofetil, with no flare-ups at the most recent follow-up.

## 3. Discussion

The index case of SISN with pan-uveitis, occurring one year after pterygium surgery, raises the question of the primary underlying pathogenic mechanism, whether autoimmunity, infection or a combination of both.

Although the specific surgical maneuver used for pterygium excision in our case was unknown, certain techniques may increase the risk of developing SISN. Adjunctive antifibrotic therapy, beta-irradiation, and excessive cautery during pterygium excision can induce local ischemia, trigger cellular apoptosis, compromise the epithelial barrier, and directly damage ocular tissue, delaying the healing process and increasing susceptibility to infection. In addition, prolonged bare sclera exposure facilitates microbial invasion and may cause enzymatic degradation of scleral collagen, ultimately resulting in scleral necrosis.^4^

Our patient had a history of cutaneous lupus erythematosus, and he was positive for p-ANCA, which suggests a broader autoimmune predisposition. Patients with autoimmune conditions undergoing scleral procedures may develop a type III hypersensitivity reaction. Such reaction is characterized by local vasculitis, HLA-DR overexpression, and neutrophil/Th cell infiltration triggered by surgical trauma or molecular mimicry, leading to ischemia and scleral necrosis.^12,13^ Tear-film abnormalities associated with altered immune system further compromise wound healing and increase collagen degradation.^4,10^ Autoimmune-associated SISN cases typically exhibit more severe scleral and ocular inflammation compared to idiopathic non-autoimmune cases.^6^ Moreover, autoimmune diseases may increase the risk of infection,^4^ and secondary superinfections have been reported in autoimmune-associated SISN associated with autoimmune conditions.^8^ On the other hand, diabetes may also be related because it exerts a pro-ischemic and pro-inflammatory environment.^9^

Co-infection with *Pseudomonas* species was highly suspected due to the presence of yellowish-green purulent exudates, subconjunctival abscess, and subretinal precipitates on OCT,^23^ along with the risk factor of prior pterygium surgery, which has been well-documented in the literature. It is notable that the culture was performed after aggressive topical antibiotic treatment, which may have led to a false-negative result. The patient improved significantly after receiving systemic antibiotics alone, without IV methylprednisolone, further supporting the role of infection in this complex index case. Similar cases of *Pseudomonas* infection following pterygium surgery with negative cultures have also been reported in a series.^24^ The conjunctival swab culture in our patient grew a small number of *Dolosigranulum pigrum*, an emerging anaerobic gram-positive ocular pathogen susceptible to beta-lactams, fluoroquinolones, and vancomycin. While previously reported in keratitis,^14–16^ conjunctivitis,^17^ and phlyctenular keratoconjunctivitis,^18^ it has not been associated with scleritis. In this index case, we believe that *D. pigrum* likely represented a contaminant, as there were no hypopyon or corneal infiltrates as described in the literature.^7,8^

In the index case, SRD was a sign mainly of posterior scleritis. Similarly, Matsuura et al. described a case of diffuse anterior scleritis accompanied by posterior scleritis and retinal detachment (RD) following pterygium excision in a p-ANCA-positive 79-year-old woman,^19^ highlighting the importance of thorough posterior segment examination. Additionally, a series by O’Donoghue et al. of 52 eyes with SISN found that 23% had evidence of secondary posterior scleritis.^9^ Concurrent hypotony due to scleral thinning is also an important contributing factor to the RD, as shown by choroidal folds in the OCT at the initial visit in our patient. In our patient, both scleral excavation and SRD showed significant resolution with improved IOP after treatment, indicating the treatmen’s effectiveness in restoring ocular structure.

Severe cases of SISN accompanied by pan-uveitis and structural complications necessitate aggressive immunosuppressive therapy to prevent vision loss and maintain the integrity of the ocular wall. Treatment may include alkylating agents like cyclophosphamide or biologic therapies such as rituximab (anti-CD20) or infliximab (anti-TNFα).^6,11^ Fidelix TS et al. reported a case of SINS following pterygium excision using the bare sclera technique in an anti-nuclear (ANA) positive female with progressive scleral thinning refractory to high-dose prednisone and combination therapy with azathioprine, as well as 12 cycles of cyclophosphamide; remission was ultimately achieved with rituximab.^20^ In managing the index case, we utilized a combination of rituximab infusion and mycophenolate mofetil. Notably, intravenous methylprednisolone was avoided due to the patien’s underlying diabetes; however, complete remission of the scleral inflammation was still achieved. Although cyclophosphamide can be used as first-line therapy, we chose rituximab due to the complex underlying systemic conditions.^6,11,20^ Additionally, oral doxycycline (100 mg/day) can be added as an adjunctive therapy for its anti-collagenolytic effect,^21^ as we did postoperatively in our patient.

Furthermore, Murthy SI et al. highlighted the benefit of surgical debridement in the management of SISN cases unresponsive to antimicrobial therapy.^7^ In accordance, a reduction of inflammation and promotion of healing were observed following surgical debridement in our patient.

In summary, SISN is a devastating and challenging ocular disorder with diverse underlying pathogenic mechanisms. Cases should not be assumed to be autoimmune in nature unless an infectious etiology has been thoroughly excluded, to avoid mistreatment and potential exacerbation of infection. Management should be tailored, and once infection is excluded, or adequately covered with antimicrobial therapy, as in this index case, aggressive immunosuppressive therapy may be required to effectively control the underlying inflammatory process.^4,22^

## 4. Conclusion

The index case report demonstrates the complex overlap between infection and autoimmunity in SISN and emphasizes the need for early biopsy or debridement when purulence or rapid scleral thinning is noted. A combination of antimicrobial therapy, aggressive immunosuppression, and timely surgical intervention may be required to prevent vision loss and ocular perforation, and to lead to successful ocular structural restoration. Rituximab infusion is a safe and effective treatment option for rapidly and robustly controlling aggressive scleral inflammation once infection has been either ruled out or appropriately managed with antimicrobial therapy.

## Abbreviations

AC: Anterior chamber
ANA: Anti-nuclear antibody
BCVA: Best-corrected visual acuity
IOP: Intraocular pressure
KPs: Keratic precipitates
OD: Right eye
OS: Left eye
p-ANCA: Perinuclear anti-neutrophil cytoplasmic antibody
RD: Retinal detachment
SD-OCT: Spectral-domain optical coherence tomography
SISN: Surgically induced scleral necrosis
SRD: Serous retinal detachment
SRF: Subretinal fluid
